# Effectiveness of artificial intelligence vs. human coaching in diabetes prevention: a study protocol for a randomized controlled trial

**DOI:** 10.1186/s13063-024-08177-8

**Published:** 2024-05-16

**Authors:** Mohammed S. Abusamaan, Jeromie Ballreich, Adrian Dobs, Brian Kane, Nisa Maruthur, John McGready, Kristin Riekert, Amal A. Wanigatunga, Mary Alderfer, Defne Alver, Benjamin Lalani, Benjamin Ringham, Fatmata Vandi, Daniel Zade, Nestoras N. Mathioudakis

**Affiliations:** 1 Division of Endocrinology, Diabetes and Metabolism, Department of Medicine, Johns Hopkins University School of Medicine, Baltimore, MD USA; 2 Department of Health Policy and Management, Johns Hopkins Bloomberg School of Public Health, Baltimore, MD USA; 3 Tower Health Medical Group Family Medicine, Reading, PA USA; 4 Division of General Internal Medicine, Department of Medicine, Johns Hopkins University School of Medicine, Baltimore, MD USA; 5 Department of Biostatistics, Johns Hopkins Bloomberg School of Public Health, Baltimore, MD USA; 6 Department of Medicine, Division of Pulmonary and Critical Care Medicine, Johns Hopkins University, Baltimore, MD USA; 7 Department of Epidemiology, Johns Hopkins Bloomberg School of Public Health, Baltimore, MD USA; 8 Reading Hospital Tower Health, Reading, PA USA

**Keywords:** Prediabetes, Health, DPP, Diabetes Prevention Program, Protocol, Randomized controlled trial, Physical activity, Artificial intelligence, AI, Digital

## Abstract

**Background:**

Prediabetes is a highly prevalent condition that heralds an increased risk of progression to type 2 diabetes, along with associated microvascular and macrovascular complications. The Diabetes Prevention Program (DPP) is an established effective intervention for diabetes prevention. However, participation in this 12-month lifestyle change program has historically been low. Digital DPPs have emerged as a scalable alternative, accessible asynchronously and recognized by the Centers for Disease Control and Prevention (CDC). Yet, most digital programs still incorporate human coaching, potentially limiting scalability. Furthermore, existing effectiveness results of digital DPPs are primarily derived from per protocol, longitudinal non-randomized studies, or comparisons to control groups that do not represent the standard of care DPP. The potential of an AI-powered DPP as an alternative to the DPP is yet to be investigated. We propose a randomized controlled trial (RCT) to directly compare these two approaches.

**Methods:**

This open-label, multicenter, non-inferiority RCT will compare the effectiveness of a fully automated AI-powered digital DPP (ai-DPP) with a standard of care human coach-based DPP (h-DPP). A total of 368 participants with elevated body mass index (BMI) and prediabetes will be randomized equally to the ai-DPP (smartphone app and Bluetooth-enabled body weight scale) or h-DPP (referral to a CDC recognized DPP). The primary endpoint, assessed at 12 months, is the achievement of the CDC’s benchmark for type 2 diabetes risk reduction, defined as any of the following: at least 5% weight loss, at least 4% weight loss and at least 150 min per week on average of physical activity, or at least a 0.2-point reduction in hemoglobin A1C. Physical activity will be objectively measured using serial actigraphy at baseline and at 1-month intervals throughout the trial. Secondary endpoints, evaluated at 6 and 12 months, will include changes in A1C, weight, physical activity measures, program engagement, and cost-effectiveness. Participants include adults aged 18–75 years with laboratory confirmed prediabetes, a BMI of ≥ 25 kg/m^2^ (≥ 23 kg/m^2^ for Asians), English proficiency, and smartphone users. This U.S. study is conducted at Johns Hopkins Medicine in Baltimore, MD, and Reading Hospital (Tower Health) in Reading, PA.

**Discussion:**

Prediabetes is a significant public health issue, necessitating scalable interventions for the millions affected. Our pragmatic clinical trial is unique in directly comparing a fully automated AI-powered approach without direct human coach interaction. If proven effective, it could be a scalable, cost-effective strategy. This trial will offer vital insights into both AI and human coach-based behavioral change strategies in real-world clinical settings.

**Trial registration:**

ClinicalTrials.gov NCT05056376. Registered on September 24, 2021, https://clinicaltrials.gov/study/NCT05056376

**Supplementary Information:**

The online version contains supplementary material available at 10.1186/s13063-024-08177-8.

## Introduction

### Background and rationale {6a}

Prediabetes currently affects more than 9% of the global population, and this figure is expected to rise to around 1 billion people in the next 20 years [1]. In the USA, it is estimated that over a third of adults have prediabetes, and of these, about 10% progress to type 2 diabetes every year, with 30–40% developing the condition within ten years [1]. Beyond being a precursor to diabetes, prediabetes is associated with elevated risks for both microvascular and macrovascular complications. Consequently, early intervention strategies are urgently needed for people with prediabetes.

Fortunately, there is strong evidence supporting lifestyle interventions, modeled on the Diabetes Prevention Program [2], focused on weight loss, healthy nutrition, and physical activity, to prevent diabetes. A meta-analysis of 16 studies conducted among diverse populations and settings showed a 41% relative risk reduction in diabetes incidence compared to usual care [3]. Multiple meta-analyses have also demonstrated that these lifestyle interventions can cause reversion to normoglycemia (from prediabetes) [3, 4].

Despite the success of the DPP, there exists glaring gaps in accessibility and uptake of the program. The USA has a mere 2147 Centers for Disease Control and Prevention (CDC) recognized DPPs, translating to an average of only one program for every 45,000 affected adults with prediabetes. This disparity is amplified by geography as only about 15% of rural counties, in contrast to about 50% of urban counties, have available DPPs [5, 6]. Compounding this accessibility issue are the low rates of DPP referrals and participation. A recent study found that only 4.2% of U.S. adults have received a DPP referral, with only 2.4% of eligible adults participating [7]. Even within the subset of referred individuals, participation rates hover at 35%, with barriers ranging from logistical challenges such as travel distance and scheduling conflicts to financial and motivational deterrents [8, 9].

To address these barriers, one approach has been the adaptation of the DPP lifestyle change program to incorporate digital health strategies. The CDC allows for DPP sessions to be delivered remotely via distance learning or through online platforms and mobile apps, which allow users to engage with materials at their own pace. Digital Diabetes Prevention Programs (d-DPPs) incorporate multiple elements of eCoaching strategies, including goal setting, self-monitoring, feedback loops, behavioral prompts and reminders, scaffolding learning, socialization, gamification, and personalization, to engage users in making meaningful lifestyle changes [10, 11]. This technology-driven approach is supported by a recent meta-analysis indicating the potential for mobile app d-DPPs to facilitate weight loss [12]. The CDC has fully recognized 16 d-DPPs (referred to as “online” programs), with several studies supporting their effectiveness [10, 13–18]. These accredited d-DPPs commonly feature tools for online meal tracking and wearable devices to monitor physical activity. Studies have demonstrated that d-DPPs can help address geographical disparities in access to the DPP [17, 19].

Much of the evidence underpinning the efficacy of d-DPPs is derived from longitudinal, non-randomized studies [13, 14]. Frequently, these studies report results based on per-protocol analyses, focusing only on participants who actively engage in or complete the program. In studies that have included a randomized control design, the control has not typically been the standard 12-month DPP. Instead, control groups often receive only single educational sessions or brief educational materials [15, 20]. Consequently, there exists a significant knowledge gap regarding the comparative effectiveness of d-DPPs against the established gold standard of the DPP. In addition, to our knowledge, none of the other commercially available d-DPPs that incorporate AI technology have no human coaching involved. By evaluating a fully-automated AI-powered d-DPP without any human coaching, this study also fills an evidence gap in understanding how well exclusively AI-driven DPPs perform against the benchmark DPP.

This paper outlines the protocol of a RCT to compare the effectiveness of a fully automated AI-powered digital diabetes prevention program (ai-DPP) to real-world human coach-based diabetes prevention programs (h-DPP) for reducing the risk of type 2 diabetes. This trial will be, as far as we are aware, the first to directly compare these two approaches head-to-head in achieving the CDC-defined type 2 diabetes risk reduction outcome.

### Objectives {7}

The overall objective of this trial is to compare the effectiveness of an ai-DPP to standard of care h-DPPs in reducing the risk of type 2 diabetes in adults with prediabetes. We hypothesize that the ai-DPP will be at least as effective as the h-DPP in attainment of the CDC’s benchmark for type 2 diabetes risk reduction.

### Trial design {8}

This study is a 12-month, parallel group, non-inferiority, open-label, multicenter RCT to evaluate whether an ai-DPP (Sweetch Health, Ltd.), comprised of a mobile app and wireless body scale, is at least as effective as a standard of care DPP. The control group participants are referred to a local CDC-recognized lifestyle change program to receive an in-person or distance learning (e.g., videoconference) based DPP. A total of 368 eligible participants have been randomized in a 1:1 ratio to receive the ai-DPP or h-DPP intervention.

We use the term effectiveness rather than efficacy as we seek to evaluate the real-world effect of both interventions. The primary endpoints across all the study aims will be assessed at 12 months, with secondary endpoints assessed at 6 and 12 months to evaluate short and longer-term effects of the intervention.

**Fig. 1:**
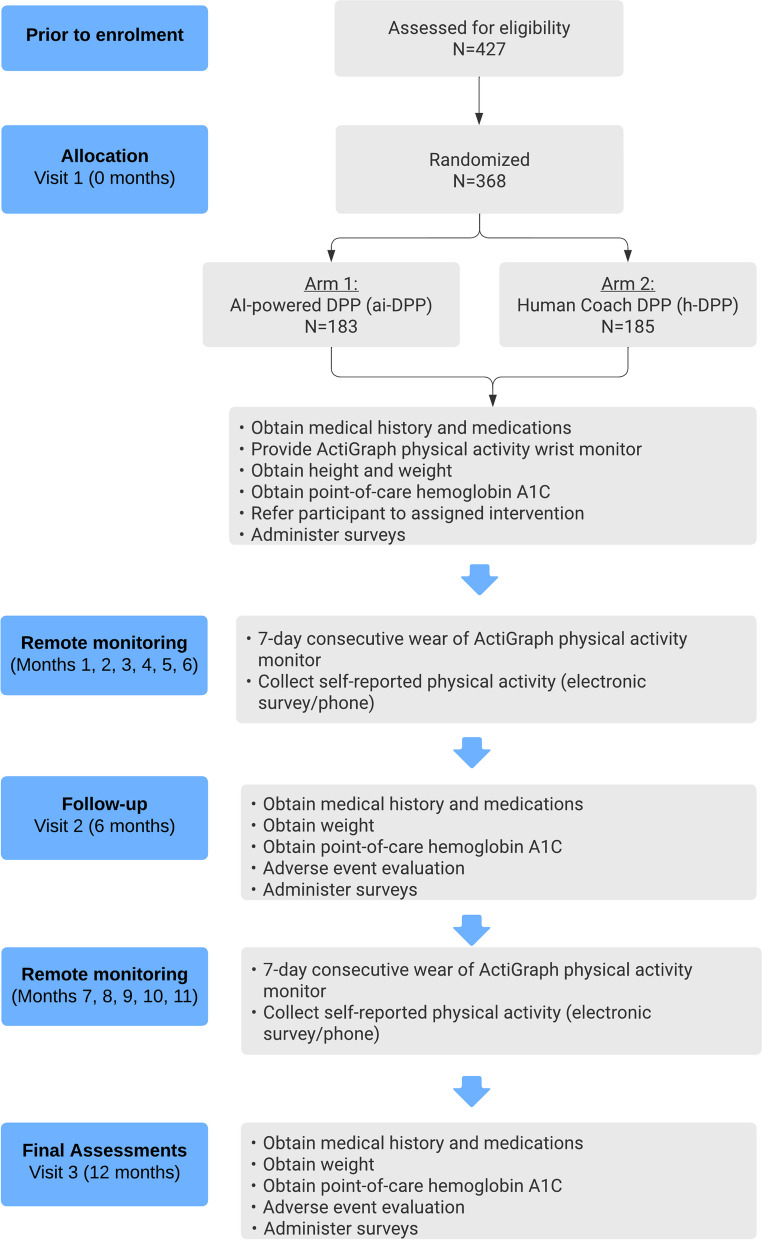
CONSORT flow diagram

This protocol is reported according to the Standard Protocol Items: Recommendations for Interventional Trials (SPIRIT) guidelines [21]. The study flow chart of enrollment, allocation, intervention, and assessment are shown in Fig. 1, and the participant timeline is presented in Table 1.

**Table 1:** Timeline of participant assessments

	**Days from enrolment**													
**Study procedures**	**0**	**7**	**30**	**60**	**90**	**120**	**150**	**180**	**210**	**240**	**270**	**300**	**345**	**365**
Study window (days)		+ 4	$\pm$ 14	$\pm$ 14	$\pm$ 14	$\pm$ 14	$\pm$ 14	$\pm$ 14	$\pm$ 14	$\pm$ 14	$\pm$ 14	$\pm$ 14	$\pm$ 14	$\pm$ 14
Study visits	x							x						x
Consent/eligibility assessment	x													
Anthropometric measurement														
Obtain height	x													
Obtain weight	x							x						x
Laboratory measurement														
Point-of-care hemoglobin A1C	x							x						x
Allocation	x													
Referral to assigned intervention (h-DPP or d-DPP)	x													
Mail Sweetch digital health kit (app and scale)		x												
Objective physical activity measurement														
7-day consecutive ActiGraph wrist monitor wear with remote CentrePoint data upload	x		x	x	x	x	x	x	x	x	x	x	x	
Questionnaires and surveys														
Demographics	x													
Medical history and medications	x							x						x
Exercise stage of change	x							x						
Self-reported physical activity	x		x	x	x	x	x	x	x	x	x	x	x	
Starting the conversation—dietary assessment	x							x						x
Apps and devices	x							x						x
NPART	x													
Acceptability								x						x
Sweetch app features (d-DPP only)								x						x
WHO-5 Well-Being Index	x							x						x
Healthcare utilization	x							x						x
Adverse event evaluation								x						x
Participant reimbursement	x							x						x

## Methods: participants, interventions and outcomes

### Study setting {9}

This trial includes two clinical sites: Johns Hopkins Hospital, an urban tertiary care academic medical center in Baltimore, MD, and Reading Hospital Tower Health, which serves a diverse urban, suburban, and rural population in Reading, PA. These institutions are both part of the Johns Hopkins Clinical Research Network, and these sites were selected for two reasons. First, both sites have CDC recognized DPPs within close proximity of their clinical research units. Second, these sites serve diverse racial and ethnic groups, which may increase the generalizability of the study findings.

### Eligibility criteria {10}

The study eligibility criteria are shown in Table 2. The study enrolled adults with overweight or obesity and a confirmed diagnosis of prediabetes. For the laboratory diagnosis of prediabetes, the most clinically available result in the past year were considered when ascertaining eligibility. If the screening A1C result at the baseline visit was not in the prediabetes range of 5.7 to 6.4%, the participant was still eligible to join the study as long as there was at least one confirmed laboratory test (most recent result) available in the previous year consistent with impaired fasting glucose, impaired glucose tolerance, or prediabetes-range A1C. These criteria are consistent with the participant eligibility criteria for the DPP as defined by the CDC [22]. For exclusion criteria, we considered factors that could affect the participant’s safety in conducting physical activity by excluding individuals with high-risk cardiovascular conditions and any other medical conditions that could affect body weight, glucose homeostasis, accuracy of A1C, as well as psychiatric or cognitive barriers.

**Table 2:** Eligibility criteria

Inclusion criteria	Exclusion criteria
∙ Male or female aged 18–75 years ∙ Laboratory evidence of prediabetes, defined as any of the following lab results, in the past year: ○ A1C of 5.7% to 6.4% ○ Fasting glucose 100–125 mg/dL ○ Plasma glucose of 140–199 mg/dL measured 2 h after a 75-g oral glucose load ∙ Body mass index (BMI) ≥ 25 kg/m^2^ (or ≥ 23 kg/m^2^ for Asians) ∙ Proficiency reading English ∙ Smartphone user (Android OS 9.0 or iOS 13.3 or newer) ∙ Plans to reside in the recruitment area (i.e., participant’s zip code of residence is within ~ 45 miles of the study recruitment site) for the next 12 months	∙ Medical conditions that hinder the adoption of moderate physical activity, as determined by the primary care clinician. We will use a modified Physical Activity Readiness Questionnaire (PAR-Q), with a particular focus on screening for potential underlying cardiac issues, to assess the appropriateness of patients for engaging in moderate physical activity (Additional file 3) ∙ Unstable cardiac disease, including myocardial infarction, heart failure, or stroke within the past 6 months, or current participation in cardiac rehabilitation ∙ Has a pacemaker, implantable cardioverter-defibrillator, or other implanted electronic device ∙ Diagnosis of aortic stenosis ∙ Diagnosis of diabetes mellitus ∙ Use of any glucose-lowering medications, weight loss medications or any systemic glucocorticoids within the previous 3 months ∙ Active malignancy of any type or diagnosed with or treated for cancer within the past 2 years. Individuals with basal and squamous cell carcinoma of the skin that have been successfully treated will be allowed to participate ∙ Diagnosis of anemia ∙ Receiving treatment for iron-deficiency anemia, vitamin B12 deficiency, or folate deficiency ∙ Hemoglobinopathy (HbS or HbC disease). Carriers of sickle cell trait (HbAS) are eligible to participate ∙ Blood transfusion in previous 4 months ∙ On dialysis or active organ transplant list ∙ Treated with erythropoietin ∙ Major psychiatric disorder (e.g., schizophrenia) or use of antipsychotic medications within the past 1 year ∙ Diagnosis of dementia or Alzheimer’s disease ∙ Diagnosed with an eating disorder (anorexia nervosa, avoidant/restrictive food intake disorder, binge eating disorder, bulimia nervosa, Pica, rumination disorder, other specified or unspecified feeding or eating disorder) ∙ Diagnosed or self-reported alcohol or substance abuse ∙ Known allergy to steel, which could affect ability to wear ActiGraph device ∙ Pregnancy or planned pregnancy in the next 12 months ∙ Participation in another clinical trial related to lifestyle management or diabetes prevention ∙ Currently attending or attended a diabetes prevention program in the previous 2 years ∙ Had bariatric surgery within the 12 months prior randomization or is planning to undergo bariatric surgery during the study ∙ Unwilling to accept random assignment

### Who obtained informed consent? {26a}

A trained research study coordinator obtained informed consent after thoroughly explaining the purpose, procedures, and potential risks of the study and addressing any questions participants might have.

### Additional consent provisions for collection and use of participant data and biological specimens {26b}

The use of participants’ data and the collection of blood samples have been outlined in the informed consent document. The point-of-care A1C test sample will be discarded after the test is collected, and there will be no storage of biological specimens.

## Interventions

### Explanation for the choice of comparators {6b}

The choice of comparators in this trial is supported by recent findings from the UK's National Health Service DPP, which demonstrated that d-DPPs can achieve weight loss outcomes at least equivalent to face-to-face interventions [23]. The control group in our study will receive the standard of care, as recommended by the American Diabetes Association, which includes a referral to a CDC-recognized DPP [24]. On the other hand, the intervention group will be engaged with a novel, ai-DPP. This program, distinct from other commercially available d-DPPs that often integrate digital tools with human coaching, operates solely on advanced AI technology, eliminating the need for human intervention.

The core of this program is the Sweetch app, which employs a sophisticated AI algorithm based on reinforcement learning. This algorithm is designed to deliver a just-in-time adaptive intervention (JITAI) [25], which is tailored to provide timely and relevant advice, track individual progress, and adapt strategies based on user feedback and receptivity. Studies on JITAIs have demonstrated their effectiveness in promoting behavior change for various goals, including weight loss and physical activity, and across various patient demographics [26–36].

The decision to use the Sweetch app in our intervention group stems from its demonstrated efficacy in a preliminary trial with adults with prediabetes, where it showed short-term success in reducing A1C levels, body weight, and increasing physical activity [37]. Among the range of commercially available digital DPPs, the Sweetch app stands out as one of the few that relies exclusively on AI technology. A recent systematic review indicated that many JITAIs are not fully automated [38]. In our selection process, we prioritized an application that embodies the full spectrum of JITAIs, characterized by its comprehensive automation and AI-driven technology.

### Intervention description {11a}

#### Intervention group

Participants in the intervention group will receive the ai-DPP comprising the Sweetch app and a wireless body weight scale from Sweetch Health, Ltd. This AI and behavioral science-based intervention excludes human coaching. Upon assignment to the ai-DPP group, participants will be mailed a digital health kit containing the app and a Bluetooth-enabled scale within 8–12 days of enrolment. The deliberate delay in sending the digital health kit was designed to allow participants to establish baseline physical activity measurements before initiating the intervention. Additionally, it replicates the real-world scenario where there may be a delay between a healthcare prescriber ordering the d-DPP and the patient receiving the product.

Referral to the Sweetch ai-DPP will be initiated through a REDCap database (see “Data management” section), triggering an automated email to the Sweetch customer service team. The referral will include the participant’s name, email address, phone number, weight, BMI, and baseline prediabetes laboratory measure(s). This procedure will allow the Sweetch team to directly contact the participant should they encounter any technical difficulties while installing the app or syncing the body weight scale. A maximum of five communication attempts will be made by the Sweetch team to register the participant in the app.

The Sweetch app collects a multitude of data inputs from the user, including historical data collected at onboarding (e.g., age, gender), real-time contextual data (e.g., location, calendar), and dynamic data (e.g., activity, weight). Data is collected both passively and actively. Passive data includes, among other data points, activity/steps, sleep/wake cycle, calendar, location, app engagement, and metadata. Actively collected data includes, among other data points, food intake, medication intake, activity not monitored passively, and weight.

The Sweetch app then analyzes this data, creates clusters, and predicts the right insights/recommendations to be presented for each user. This user state is then examined in the context of the COM-B framework [39]. The individual’s needs and challenges are identified (e.g., oversubscribed calendar identifies opportunity as the challenge) and personalized notifications that employ behavioral change techniques (BCTs) are selected to address an individual’s challenge. A range of BCTs are employed by the Sweetch app, including goal-setting, action planning, feedback on behavior, self-monitoring of behavior and outcomes, information about health consequences, prompts/cues, habit formation, and more [40].

Sending of these contextual messages activates the app’s reinforcement learning (RL) engine. The RL engine assesses whether the intervention leads to action and accordingly creates additional messaging depending on response. This loop is perpetually updated based on the individual’s current context and real-life circumstances.

Participants have the flexibility to toggle push notifications on or off within the Sweetch app. They cannot, however, modify the frequency or timing of notifications, as these are dynamically generated using AI technology, and by adapting to the participant’s behavioral patterns and progress. Supplementary education may be provided via emails in addition to the in-app lessons.

#### Control group

Participants assigned to the h-DPP arm will receive standard care, which includes a referral to a 12-month CDC-recognized lifestyle change program. The study team will arrange for the participant to join a participating h-DPP at the soonest available cohort start date. To ensure the h-DPP aligns with the study’s schedule, randomization was halted whenever no upcoming h-DPP cohorts were available within the next 30 days, often due to holiday periods. While the seasonal availability of cohorts may hinder h-DPP participation, halting randomization to coincide with DPP cohort starts was necessary to synchronize study activities and outcome measurement with the intervention's actual implementation. These interruptions might inadvertently lower barriers to h-DPP engagement and increase participation, potentially skewing the results against our hypothesis by reducing the differences between control and intervention groups. However, aligning the study period with the timing of the intervention is crucial for accurately gathering data on participant engagement in h-DPPs.

The referral process to the h-DPP will be initiated through a REDCap database, automatically triggering an email to the local h-DPP coordinator. The REDCap referral will include the participant’s name, email address, phone number, weight, BMI, and baseline prediabetes laboratory measure(s). This procedure will allow the local h-DPPs to initiate outreach in accordance with their standard protocols. As with the d-DPP group, a maximum of five attempts will be made by the h-DPP to enroll the referred study participants.

Eligible DPPs within a 45-mile radius of the study sites (Baltimore, MD, and Reading, PA) will be identified through the CDC’s Recognized Lifestyle Change Program Website, which hosts a registry of recognized programs [41]. For this trial, to qualify, local h-DPPs must satisfy the following criteria:

- Have either preliminary or full recognition status from the CDC
- Offer in-person or synchronous distance learning modalities (via video conferencing)
- Consent to sharing attendance and DPP outcome data (e.g., weight), in accordance with CDC reporting requirements, using the study team’s electronic data capture form.

To attain preliminary or full recognition, local h-DPPs must adhere to the CDC’s Diabetes Prevention Recognition Program Standards and Operating Procedures. Sessions are delivered by trained lifestyle coaches and include a minimum of 16 in the first 6 months (core phase) and 6 in the last 6 months (core maintenance phase). Each program follows the CDC-approved PreventT2 curriculum. Core sessions are held weekly for the first 6 months, then typically bi-weekly for the core maintenance sessions in months 7–12.

The CDC recognizes several modalities for DPP, including in-person, distance learning, and online. Distance learning refers to both synchronous and asynchronous delivery using video and phone conferencing. In synchronous delivery, instruction is conducted face-to-face in real-time. Synchronous delivery most closely resembles a traditional classroom, despite the participants being located remotely. It requires an organized timetable and an instructor to be present. Participants typically interact with the instructor and each other. In asynchronous delivery, instruction is self-paced. Participants access course materials on their own schedules and are not required to be together at the same time. Delivery technology includes video and audio recordings, discussion board forums, e-mail, and self-directed print materials. Hybrid, or blended, learning is when synchronous and asynchronous technologies are combined.

Among these CDC-recognized delivery modalities, for participants randomized to receive the h-DPP, local DPPs will only be permitted to use in-person, synchronous distance learning, or combined modality to avoid contamination with the d-DPP intervention. The online-only modality will not be permitted. Online platforms often use wireless weight tracking, physical activity trackers, and social support and engagement tools that would introduce contamination with the study intervention. In addition, asynchronous distance learning alone will not be permitted, as it will be practically challenging to track participant engagement in these programs.

The in-person delivery of these local h-DPPs occurs in various settings (hospital outpatient, primary care, community, church). Programs may use any video conferencing platform (e.g., Zoom, WebEx, Google Meet) to deliver synchronous distance learning.

The COVID pandemic, which occurred during this trial, posed significant challenges to the traditional in-person delivery of the DPP, leading the majority of local programs to shift towards remote learning via video conferencing. Importantly, a study of DPP during the COVID pandemic found that weight losses achieved through remote and digital interventions were greater than those previously achieved through face-to-face interventions, suggesting that the transition to a remote format of DPP not only addressed the challenges posed by the pandemic but also enhanced the effectiveness of the program in terms of weight loss outcomes [17]. In many ways, the transition to distance learning DPP has helped to remove many of the barriers to participation in h-DPPs by removing the travel and scheduling barrier. Although this could increase engagement in the h-DPP and thereby narrow the difference in effectiveness between the two arms, this study still addresses whether AI technology can perform as well as a human coach, even if the human coaching is largely remote. These findings are more likely to generalize to h-DPPs that are predominantly offered through distance learning compared to in-person.

Given that these programs admit participants at varying intervals and on a rolling basis, it will be explained to participants that once they begin a program, they are expected to remain in that program for at least the core phase’s duration (months 1–6). Following this core phase, they may have the option to switch to an alternative h-DPP if they choose to do so. Nevertheless, participants will be strongly encouraged to complete the entire program within the same h-DPP for the best outcomes.

### Criteria for discontinuing or modifying allocated interventions {11b}

Given the focus of this study on increasing physical activity and promoting a healthy diet and weight loss, we do not anticipate any serious adverse events that require discontinuation of the study intervention. The study team will only withdraw a participant if any clinical adverse event, laboratory abnormality, or other medical condition or situation occurs such that continued participation would not be in the best interest of the participant.

For participants who do not engage with the study intervention, whether randomized to the d-DPP or h-DPP, all efforts will be undertaken by the research coordinator to retain the participant in the study for effectiveness assessment. For instance, if a participant requests to withdraw from the study due to dissatisfaction with any aspect of the interventions, our research coordinators will offer the option to remain in the study, if they are willing to complete the required study visits. Participants who sign the informed consent form, are randomized, and receive the study intervention, and subsequently withdraw, or are withdrawn or discontinued from the study, will not be replaced.

### Strategies to improve adherence to interventions {11c}

Presently, some local DPPs participating in this trial seek reimbursement from insurance companies for the services they provide to their beneficiaries. To encourage participation in the trial, we will cover all costs of the DPP for all study participants who are randomized to a local DPP. Participants also receive gift cards upon completing study visits and monthly actigraphy wear periods. However, it is important to note that since this is a real-world effectiveness study, there may be various barriers affecting adherence to both AI and human-coach-based DPPs. As such, the study team will not aggressively intervene to ensure participant adherence to their assigned intervention, recognizing the need to observe real-world adherence patterns.

Since access to the h-DPP and ai-DPP are controlled by the study investigators, it is not possible for a study participant to cross-over to the other arm. Nonetheless, we cannot guarantee that participants assigned to the h-DPP would never receive human coaching or that they could resort to using mobile apps to support their lifestyle changes; similarly, it is possible that participants assigned to the ai-DPP would choose not to use the app and resort to use of human coaches outside of the DPP (e.g., local gym).

### Relevant concomitant care permitted or prohibited during the trial {11d}

Participants will be expected to adhere to their assigned intervention but are not limited in using additional tools to support healthy lifestyle behaviors. We will collect information about the usage of apps and devices for tracking weight, physical activity, and nutrition during the 6- and 12-month study visits for both groups. Participation in other trials or programs related to nutrition, weight, or diabetes is not allowed. It is anticipated that some participants from either group might transition from prediabetes to type 2 diabetes during the study. If a primary care provider decides to initiate glucose-lowering medication(s) for a study participant, the participant will remain eligible to continue, and all new medications that are initiated for glycemic control will be recorded.

### Provisions for post-trial care {30}

We do not plan for any post-trial care. Any adverse events reported by participants during the trial will be promptly addressed. Post-trial care or follow-up is not expected.

### Outcomes {12}

#### Primary outcome

The trial is designed as a non-inferiority study to evaluate whether the ai-DPP is at least as effective as h-DPPs in helping participants attain the CDC’s benchmark for reducing the risk of type 2 diabetes. This benchmark is defined as achieving one or more of the following criteria at 12 months:

- A minimum of 5% weight loss
- A minimum of 4% weight loss combined with at least 150 min per week of physical activity
- A minimum reduction of 0.2 points in A1C level (applicable only to participants with an A1C result between 5.7% and 6.4% at the baseline study visit)

The selection of the primary endpoint aligns with the current CDC standards for full recognition of DPP programs, effective as of May 2021. Participants who become pregnant during the study or whose A1C reaches the diabetes range (i.e., 6.5% or greater) at either the 6- or 12-month study visit will be deemed as not meeting the primary endpoint. Consistent with the CDC guidelines, which exclude pregnant individuals and those with diabetes-range A1C from outcome evaluations, our trial will deem these participants as not meeting the endpoint but will continue to include these participants in the data analysis to preserve the integrity of the randomization process.

Weight measurements will be taken using a medical digital scale with precision to the nearest 0.1 kg. Participants will be instructed to wear lightweight clothing, remove their shoes, and empty their bladders before the weight measurement.

A1C levels will be assessed using FDA-approved, CLIA-waived, and NGSP-certified devices, such as the AfinionTM 2 Analyzer or A1CNow + test kit, administered by certified and trained study coordinators.

For monitoring physical activity, participants will receive an ActiGraph accelerometer model GT9X or CentrePoint® Insight Watch (CPIW), both of which have received FDA clearance (ActiGraph, LLC, Pensacola, FL, USA), during their initial visits. Participants will be required to wear the device on their non-dominant wrist for seven consecutive days upon enrolment. Then, at 1-month intervals throughout the study, the participant will be asked to wear the device for seven consecutive days (i.e., approximately 1 week on, 3 weeks off), for a total of 12 wear periods. Activity counts will be recorded at frequencies of 30 Hz (GT9X) and 32 Hz (CPWI) and then aggregated into 60-s epochs. To assess activity levels at moderate to vigorous physical activity (MVPA) intensities, we will process the data using the most recently validated cutoff points for adults.

To better distinguish exercise from all-purpose MVPA, only consecutively occurring MVPA lasting 10 min or more will be summed. We will calculate the sum of consecutively occurring active minutes lasting 10 min or more for each monthly wear period and then compute an average over the months 1–11 of the study (excluding the baseline measurement). Non-wear will be assumed to be minutes not reaching MVPA, otherwise defined as physical inactivity. Consequently, participants will remain eligible for the study even if they do not complete each monthly physical activity assessment.

#### Secondary outcomes

Secondary outcomes will include:

- A1C level changes: change in A1C from baseline to 6 months, baseline to 12 months, and 6 months to 12 months.
- Incidence of type 2 diabetes: calculation of the proportion of participants meeting the A1C criterion (≥ 6.5%) for type 2 diabetes. A1C will serve as a proxy for diabetes incidence, recognizing that additional tests are typically necessary for confirmation.
- Weight changes: evaluation of absolute and percentage weight changes at baseline, 6 months, and 12 months.
- Changes in physical activity: analysis of changes in average minutes per week of physical activity from baseline to 6 months and baseline to 12 months.
- Objective vs. subjective physical activity correlation: across both groups, a comparison between self-reported and objectively measured activity will be performed. Self-reports will be collected at 12 intervals using the IPAQ-SF after each 7-day ActiGraph wear period (refer to Additional file 1).
- Engagement levels: a comparative analysis of engagement levels between ai-DPP and h-DPP will be done to evaluate the association between engagement and clinical outcomes. Engagement levels for the h-DPP will be based on attendance and of the ai-DPP will be based on app use metrics. Recognizing that there are fundamental differences in engagement metrics between the groups, we will attempt to normalize engagement measures between the two arms (i.e., using percentile ranking) to enable pooled analyses of engagement with clinical outcomes across both groups.
- Program completion rate: determination of the percentage of participants who successfully complete the program. For h-DPP, CDC-defined completers attend at least eight sessions within months 1–6, spanning at least 9 months. In the ai-DPP arm, completion entails at least 8 weeks of engagement in months 1–6, with a time span of at least 9 months between app installation and the last week of engagement.
- Acceptability: comparative assessment of intervention acceptability, including satisfaction, utility, motivation, and more, using a 32-item questionnaire we developed at 6 and 12 months.
- Well-being score changes: measurement of well-being using the five-item WHO-5 index questionnaire at baseline, 6 months, and 12 months [42].
- Predicting response to ai-DPP vs. h-DPP: we will evaluate whether participant characteristics influence success with either of the two lifestyle change approaches. We hypothesize that younger, tech-savvy adults may be more likely to succeed with ai-DPP compared to older less tech-savvy adults, who may benefit from the h-DPP. We will use an adapted 36-item NPART survey [43] to assess need for social interaction and digital skills. If non-inferiority of the intervention is shown, NPART could help identify optimal DPP modality for individual patients.
- Cost-effectiveness: comparative evaluation of the cost-effectiveness of the two interventions over a lifetime horizon employing a Markov model. This model will derive parameters from trial outcomes and published literature to establish the incremental cost-effectiveness ratio. See “Statistical analysis” section for more details.

### Participant timeline {13}

The study consists of three study visits: baseline, 6 months, and 12 months. In addition, 7-day consecutive blinded actigraphy assessments occur at baseline and once per month throughout the trial. The participant timeline is shown in Table 1.

### Sample size {14}

Under a 1:1 randomization design, with a significance level (alpha) set at 0.05 and 80% power, and assuming that 50% of participants achieve the primary outcome in both study arms, it is necessary to enroll 138 participants in each of the two arms, resulting in a total study sample size of *n* = 276.

Considering a conservative attrition rate of 25% at the 12-month mark, the adjusted sample size is increased to 184 participants per group, totaling 368 participants. This adjustment ensures that the minimal necessary analyzable sample of 276 is retained.

If there is indeed no difference between the h-DPP and ai-DPP, then having 276 participants allows us to be 80% confident that the upper limit of a one-sided 95% confidence interval will exclude a difference in favor of the h-DPP by more than 15%. Although it is acknowledged that the probability of success (*π*_*s*_) is likely to fall in the 30–40% range, we used 50% in the non-inferiority sample size calculations. This choice was made because it results in the largest necessary sample size among all options for *π*_*s*_, making the study robust in terms of statistical power across various values for* π*_*s*_.

### Recruitment {15}

Enrolment for this study has completed. We employed a variety of recruitment strategies to achieve our target sample size and ensure a broad representation of participants. The recruitment methods for participants who were screened, deemed ineligible, and ultimately enrolled are provided in Additional File 2. Direct messaging through electronic health record portals (Epic MyChart or Epic MyTower) to individuals meeting the eligibility criteria proved to be the most effective method for enrolling participants, accounting for 45.9% of the total. This was followed by BuildClinical (16.9%), a clinical trial recruitment firm utilizing machine learning algorithms and social media analytics to identify and engage potential participants. Other successful strategies included direct referrals from healthcare providers (13.6%), social media advertisements on Facebook (5.4%), and our study's website (4.3%). Less prevalent methods comprised flyers (1.4%), Clinicaltrials.gov (0.5%), and postcards (5.4%). Notably, participants who showed interest via the study website and postcards were more likely to be ineligible compared to those recruited through other methods.

## Assignment of interventions: allocation

### Sequence generation {16a}

The randomization sequence was generated using block randomization techniques, with block sizes randomly assigned as 2, 4, or 8, and allocated to two strata based on baseline A1C levels (either 5.7% to 6.0% or 6.1% to 6.4%) and recruitment site (either Johns Hopkins or Reading). This stratification was done to ensure a balanced distribution in each group with regard to glycemic control and recruitment location. It is important to highlight that a higher A1C level at baseline is a significant predictor of diabetes development and the potential need for glucose-lowering medication.

### Concealment mechanism {16b}

The allocation sequence was implemented via a REDCap interface that concealed the sequence until the interventions were assigned to a participant.

### Implementation {16c}

The allocation table was created by the study statistician and uploaded to REDCap. This sequence was executed through a database interface using REDCap, which keeps the sequence concealed until a participant is assigned to a group. The study coordinator informed participants of their treatment assignment after completing all baseline visit questionnaires. This process ensured the integrity of the allocation sequence and maintained blinding until the participant's assignment was disclosed.

## Assignment of interventions: blinding

### Who will be blinded {17a}

While participants and research coordinators are aware of the group assignments, the data analysis will be conducted by the study statistician, who will remain blinded throughout the analyses involving the primary endpoint.

### Procedure for unblinding if needed {17b}

Not applicable as no blinding was used for group assignment in this trial.

## Data collection and management

### Plans for assessment and collection of outcomes {18a}

Data collection will be diligently managed by our study staff under the supervision of the site Principal Investigator (PI), ensuring accuracy, completeness, and timeliness. The following procedures will be employed for data collection:

- Baseline measures: we will obtain fingerstick A1C measurements, record height, and body weight and collect demographic information, including age, sex, race, ethnicity, marital status, educational attainment, and employment status. Participants will also be asked to complete a set of questionnaires covering topics such as general health, overall well-being, medical conditions, current medications, socioeconomic factors, physical activity, nutrition, and technology usage.
- 6- and 12-month visits: during these visits, we will record fingerstick A1C levels and body weight. Participants will be asked to complete the initial set of questionnaires, in addition to supplementary questionnaires addressing their satisfaction with both the ai-DPP and h-DPP interventions.
- Actigraphy monitoring: throughout the 12-month duration of the study, participants will be provided with an Actigraph wrist monitor, which they will wear continuously for seven consecutive days upon enrollment and approximately once a month thereafter. To minimize direct team contact and potential impacts on DPP engagement, automated reminders will be sent to participants via text or email prior to each wear period. In addition to wearing the device, participants will be requested to complete monthly self-reported physical activity questionnaires.

### Plans to promote participant retention and complete follow-up {18b}

To enhance participant retention and facilitate complete follow-up, we have implemented several measures to minimize the burden on participants:

- Streamlined assessments: we have combined the screening and baseline assessments into a single visit. This consolidation is made possible by utilizing point-of-care A1C measurements for prediabetes diagnosis, eliminating the need for fasting labs and subsequent visits.
- Flexible home study visits: at the Johns Hopkins site, participants are offered the convenience of home study visits to increase flexibility and reduce the need for travel.
- Financial incentives: participants will receive $40 for each successfully completed study visit and an additional $10 for each 7-day period of ActiGraph wear time, totaling 12 measurement periods. This provides the potential for participants to earn up to $240 for their participation in the entire study. Moreover, we will cover all parking expenses for each visit.
- Reminders: participants will receive timely reminders for study visits and monthly actigraphy. These reminders will be delivered using the participant’s preferred method, as indicated in their communication preferences.
- Addressing barriers: during the baseline visit, we will engage participants in discussions about potential or anticipated barriers to attending follow-up research visits, allowing us to proactively address these concerns.
- Appreciation: as a token of our appreciation, enrolled participants will receive virtual holiday and birthday cards via email, recognizing their contribution to the study and fostering a sense of community and gratitude.

### Data management {19}

Study data from research visits will be collected and managed using REDCap electronic data capture tools hosted at Johns Hopkins University [44, 45]. REDCap (Research Electronic Data Capture) is a secure, web-based software platform designed to support data capture for research studies, providing (1) an intuitive interface for validated data capture; (2) audit trails for tracking data manipulation and export procedures; (3) automated export procedures for seamless data downloads to common statistical packages; and (4) procedures for data integration and interoperability with external sources. Study staff will be responsible for entering data in real-time using electronic case report forms (eCRFs) within the REDCap platform.

In the d-DPP arm, data will be transmitted in real-time from the participant’s smartphone to a secure server. All data communication between participant devices and the server will be encrypted using secure sockets layer (SSL) certificates before storage. Data transfers between smartphones and the server will be encrypted and accessible exclusively through SSL with 128-bit encryption across all channels, both for transmission from the smartphone to the server and from the server to the database. Data access will be restricted to authorized users from Sweetch Health, Ltd. and the study staff, with access controlled through unique usernames and passwords. Every instance of data access will be logged, ensuring accountability and security.

For the actigraphy data, we will utilize the ActiGraph CentrePoint system. This system automates the collection of actigraphy data from the deployed activity monitors and ensures the protection and easy retrieval of source data records. Authentication is required to access the system web portal for viewing or retrieving data. The CentrePoint system and data storage infrastructure are hosted within the secure environments of Amazon Web Services (AWS) and Microsoft Azure. These vendors provide ActiGraph with infrastructure as a service, including robust security measures, data backups, and other essential data center services. Source data captured from activity monitors is stored in both Amazon’s Simple Storage Service (S3) and Relational Data Storage (RDS) systems, distributed across the USA. Web services and application interfaces are deployed within the Microsoft Azure cloud platform to establish a secure framework for our public-facing cloud services.

Each participant will be assigned a unique study identification number within the CentrePoint system. No personally identifiable information will be entered or collected within the CentrePoint platform, ensuring participant privacy and data security. Raw data will be exported from CentrePoint as GT3X file format. The ActiLife software (version 6.13.5) will be used to convert the GT3X into a CSV file, which will be used for the comprehensive analysis of all acquired actigraphy data.

### Confidentiality {27}

To uphold confidentiality throughout the research activities, we will ensure that all study proceedings take place in as private a setting as feasible. All study documentation, generated data, and any other related information will be maintained under strict confidentiality protocols. No information pertaining to the study or its data will be disclosed to any unauthorized third party without obtaining prior written approval from the sponsor.

Each participant will be assigned a unique trial identification number, and all collected data will be securely stored within the REDCap system. Access to this data will be restricted solely to the study team, utilizing encrypted computers to maintain the highest level of data security.

Upon the conclusion of the study, all records will continue to be safeguarded in a secure location for a duration specified by the reviewing Institutional Review Board (IRB), institutional policies, or sponsor requirements, ensuring ongoing protection of participant information and data integrity.

### Plans for collection, laboratory evaluation and storage of biological specimens for genetic or molecular analysis in this trial/future use {33}

Not applicable. No biological specimens will be collected in this trial.

## Statistical methods

### Statistical methods for primary and secondary outcomes {20a}

This clinical trial will be conducted under a common protocol, and data analysis will involve pooled data from both study sites. A biostatistician will oversee the data analysis process. The following statistical approaches will be employed:

1. Exploratory data analysis: initial data exploration will include the examination of outliers, characterization of the distributions of continuous and categorical variables, and monitoring of missing data. Mean, median, and frequency counts will be utilized to summarize baseline characteristics that are measured on a continuous scale. Proportions and frequency counts will be employed for categorical measures.
2. Baseline statistical differences: univariate analysis will be conducted to assess baseline statistical differences between the treatment groups. Continuous measures will be evaluated using unpaired *t*-tests or Wilcoxon signed-rank tests, while categorical measures will be assessed using chi-square tests or Fisher’s exact tests, as appropriate.
3. Primary effectiveness endpoint: the primary effectiveness endpoint will be analyzed using an intention-to-treat (ITT) approach. Logistic regression models will be employed, with the attainment of the primary endpoint serving as the dependent variable and treatment group as the primary exposure variable. Covariate-adjusted models will be constructed, incorporating variables identified as statistically significant differences between the two treatment groups during univariate analysis, both at baseline and over the study period. This adjustment will include variables related to the use of medications that could influence blood glucose (e.g., antihyperglycemics, steroids) and incident diabetes.
4. Secondary endpoints: secondary endpoints will also be analyzed in accordance with the ITT principle. Each of the individual outcomes within the composite primary endpoint will be analyzed as secondary endpoints at both the 6- and 12-month time points. Similar to the primary endpoint, each outcome will be analyzed using logistic regression models that include a random intercept to account for within-person outcomes clustering. Additionally, changes in A1C, absolute weight change, and percentage weight change will be analyzed as continuous measures employing linear regression models, also including a random intercept to account for within-person outcomes clustering. Various physical activity measures, including average minutes per week of physical activity (light, moderate, intense), MET-hours per week of physical activity, and average daily step counts, will also be treated as continuous measures. Depending on the distributions of responses from the acceptability questionnaire (Likert scale questions), these will either be dichotomized (acceptable vs. non-acceptable) and analyzed using logistic regression or kept as ordinal multicategorical and analyzed with ordinal logistic regression.
5. Cost-effectiveness analysis: cost-effectiveness analysis will be conducted for both the 12-month and lifetime horizons. In the 12-month analysis, cost and effect differences between participants in the two arms will be assessed. For the lifetime horizon, a Markov model will be constructed, with model parameters derived from trial results and published literature. Cost savings associated with intermediate endpoints (e.g., percentage weight loss reduction, A1C reduction, increased physical activity levels) will be estimated. Both analyses will adopt a health system perspective, discounting future costs and effects at a rate of 3%. In the lifetime horizon analysis, a Markov model will simulate hypothetical patients exposed to either a d-DPP or h-DPP, encompassing health states reflective of prediabetes and diabetes, including normal glucose tolerance, prediabetes, type 2 diabetes mellitus, and death. Transition probabilities between these health states will be calculated based on trial data and published literature, with diabetes incidence estimated using changes in A1C as a proxy. Life table estimates from the CDC will determine probabilities of death in all health states. Healthcare costs will encompass both formal healthcare costs and informal healthcare costs, estimated using healthcare resources utilized multiplied by resource prices. Unit prices will be derived from public databases and Medicare fee schedules. Cost data, including intervention costs, will be provided by Sweetch Health, Ltd. Costs for the human coach intervention in the h-DPP arm will be estimated using the Medicare Diabetes Prevention Program (MDPP) billing and fee schedule. The analysis will employ quality-adjusted life years (QALYs) as the measure of effect, with QALYs calculated based on time spent in specific health states multiplied by utility weights. Utility weights will be derived from published literature. Time spent in each health state for the 12-month analysis horizon will be estimated using trial data, while the lifetime horizon will involve assigning utility weights to health states and utilizing the Markov model to determine time spent in each state over a patient’s lifetime. Incremental cost-effectiveness ratios (ICERs) between the two arms will be calculated, with results compared to standard willingness-to-pay thresholds for similar public health programs in the US. Cost-effectiveness will be established if the ICER falls below the willingness-to-pay threshold. Both univariate and probabilistic sensitivity analyses will be conducted by varying key transition, utility, and cost parameters. The results of the probabilistic sensitivity analysis will be used to construct a cost-effectiveness acceptability curve.

### Interim analyses {21b}

There is no planned interim analysis for this trial.

### Methods for additional analyses (e.g., subgroup analyses) {20b}

In addition to the primary analysis, several additional analyses will be performed to explore various aspects of the study and provide a comprehensive understanding of the results:

1. Per-protocol analysis: a per-protocol analysis will be conducted using the population of program completers in each group.
2. Sensitivity analyses by study site: this trial will enroll participants from two sites, which serve patient populations with distinct sociodemographic characteristics. Sensitivity analyses will be conducted to account for the potential impact of the study site on the outcome measures. These analyses will assess whether there are differences in treatment effects or responses to treatment based on the study site.
3. Exploratory analyses within treatment arms: exploratory analyses will be undertaken within the h-DPP and ai-DPP arms to identify factors associated with success in each group. While not the primary focus of this trial, a single-arm analysis of the h-DPP will be conducted to explore potential differences in outcomes between the study sites.
4. Sensitivity analyses for primary endpoint: during the course of this study, participants may receive antihyperglycemic medications, such as metformin, or weight loss drugs, such as glucagon-like peptide 1 receptor agonists, even if they do not have a diabetes diagnosis. The use of these medications could potentially skew the results by causing favorable reductions in A1C or weight. To address this issue, we will not only adjust for the use of these medications in our analyses but also perform a sensitivity analysis. In this analysis, any participants using antihyperglycemic or weight-loss medications will be considered as not having met the primary endpoint, regardless of their achievement of any specified criteria within our composite endpoint. This sensitivity analysis will help to assess the true effectiveness of the intervention by isolating its impact from that of any medication use.

### Methods in analysis to handle protocol non-adherence and any statistical methods to handle missing data {20c}

In line with the intention-to-treat principle, the analysis will adhere to the following strategies to handle protocol non-adherence and address missing data:

1. Intention-to-treat analysis: the results of all study participants will be assessed based on their initial randomization, regardless of whether they adhered to their assigned treatment or encountered protocol violations or deviations. Drop-out reasons and protocol deviations will be evaluated across all groups, and the characteristics of individuals involved will be compared between the intervention and control groups. Data will be included in the analysis up to the point of drop-out. However, for analyses of effectiveness endpoints, participants who withdraw from the study or withdraw consent will be excluded.
2. Handling missing data: it is expected that there will be no missing data for A1C and weight measurements among study completers, as these measures are collected by study coordinators during study visits. In contrast, missing data may be more prevalent for the physical activity outcome variable since it relies on participants wearing ActiGraph devices at 1-month intervals. Missing data (i.e., non-wear time) will be presumed to represent physical inactivity, and we do not anticipate differences in missingness by treatment assignment. Participants will remain eligible for the weight loss and A1C outcomes even if they are missing the physical activity data.
3. Assessment of missing data patterns: missing values will be quantified, and their patterns will be assessed to determine if they are missing at random. Means among different observed patterns of missingness will be compared to detect non-random missing data.
4. Multiple imputations: if the pattern of missingness suggests that the data are not missing at random, multiple imputations will be employed. This statistical technique will be used to estimate the treatment effect while appropriately modeling the variability in the data. It helps mitigate bias and ensures robustness in the analysis by imputing missing values with plausible estimates based on observed data.

### Plans to give access to the full protocol, participant-level data and statistical code {31c}

The full study protocol, de-identified datasets analyzed, and statistical code will be available from the corresponding author on reasonable request and with the proper regulatory permissions.

## Oversight and monitoring

### Composition of the coordinating center and trial steering committee {5d}

The coordinating center for this trial is led by Dr. Nestoras Mathioudakis at the Johns Hopkins School of Medicine, who serves as the Principal Investigator. In the preparatory phase, Dr. Mathioudakis convened monthly meetings with co-investigators, transitioning to quarterly meetings during the active phase of the trial.

The day-to-day operations of the study are overseen by the project manager and research coordinators. Their roles encompass various key responsibilities, including participant recruitment management, supervision of data collection procedures, submission of protocol changes, and collaboration with the Institutional Review Board (IRB) for necessary revisions. They also monitor data reports and ensure strict compliance with all mandatory training requirements.

Additionally, the lead project manager takes charge of developing the study’s data tracking system and establishing robust data validation procedures to maintain the integrity and accuracy of the trial’s data.

### Composition of the data monitoring committee, its role and reporting structure {21a}

A three-member Data Safety and Monitoring Board (DSMB), consisting of a clinical investigator, a public health researcher, and a biostatistician, have been established. The DSMB is entirely independent of the study’s conduct and is free from any conflicts of interest. Regular DSMB meetings will be conducted, with a minimum frequency of once per year, to thoroughly evaluate safety and efficacy data for both study arms.

The DSMB will operate in accordance with the guidelines set forth in an approved charter. This charter was drafted and reviewed during the inaugural organizational meeting of the DSMB, ensuring transparency, accountability, and adherence to best practices in monitoring the study’s progress and safeguarding participant well-being.

### Adverse event reporting and harms {22}

This lifestyle intervention study is considered low-risk, and we do not anticipate any adverse events directly attributable to participation. However, we will strictly adhere to the Johns Hopkins Organizational Policy on Prompt Reporting of Reportable Events, which includes unexpected deaths and unanticipated problems involving risks to subjects or others.

Any adverse events are expected to be reported initially to research coordinators during scheduled study visits or through follow-up communications. These events will be comprehensively documented in the participant’s electronic case report form, which includes details such as the date of onset and resolution, a description of the problem, the preferred term for the adverse event, and the perceived grade of the event using the following grading system:

- Grade 1: mild, asymptomatic, or mild symptoms; clinical or diagnostic observations only; no intervention required.
- Grade 2: moderate, requiring minimal or local intervention.
- Grade 3: severe or medically significant but not immediately life-threatening, necessitating hospitalization or prolongation of hospitalization; disabling; limits self-care activities of daily living.
- Grade 4: life-threatening consequences requiring urgent intervention.
- Grade 5: death related to the adverse event.

In the event of a perceived Grade 4 or higher adverse event (indicating life-threatening consequences or the need for urgent intervention, or death related to an adverse event), the Research Coordinator will promptly notify the Principal Investigator (PI) and Project Manager via phone or text message. The PI will then assess the severity and potential relationship of the adverse event to the intervention.

The study team will maintain continuous contact with affected participants until the issue has been satisfactorily resolved or stabilized. Furthermore, if it is determined that five Grade 3 adverse events are “probably related” to the study intervention, the PI will immediately inform the Institutional Review Board (IRB), the Data Safety and Monitoring Board (DSMB), and the NIH program officer. This proactive approach ensures that participant safety remains paramount throughout the study.

### Frequency and plans for auditing trial conduct {23}

The project management team, comprising the study coordinator(s), Principal Investigator (PI), and other essential personnel, will convene weekly meetings to comprehensively assess trial progress, recruitment status, participant inquiries or issues, and other pertinent matters.

In addition to these weekly meetings, the complete project team, including all coinvestigators, will gather on a regular basis to conduct in-depth evaluations of trial advancement, participant concerns, and to conduct thorough reviews of trial protocols and procedures. This broader team collaboration will ensure that all aspects of the study are rigorously monitored and optimized.

To maintain data integrity and adherence to established protocols, the PI and project manager will conduct data audits at twice-monthly intervals. These audits will leverage the built-in report functionality in REDCap to identify missing data or potential outliers. This internal auditing process will serve as a proactive measure to promptly identify and address any data discrepancies or issues.

Furthermore, the study team is committed to maintaining transparency and accountability by providing annual progress reports to both the institutional review board and the National Institute of Diabetes and Digestive and Kidney Diseases (NIDDK), who serves as the study sponsor. This regular reporting mechanism will help ensure that the trial is conducted in adherence to established protocols and in accordance with ethical and regulatory standards.

### Plans for communicating important protocol amendments to relevant parties (e.g. trial participants, ethical committees) {25}

In the event of any necessary modifications to the trial design, including changes related to trial eligibility criteria, a comprehensive review will be conducted by the institutional review board. Upon receiving approval from the institutional review board for these proposed changes, the protocol amendments will be promptly and accurately updated on ClinicalTrials.gov. This transparent and proactive approach ensures that the most current and accurate trial information is readily available to the public, trial participants, and relevant ethical committees.

### Dissemination plans {31a}

This study is committed to adhering to the NIH Data Sharing Policy, the Policy on the Dissemination of NIH-Funded Clinical Trial Information, and the Clinical Trials Registration and Results Information Submission rule. The trial has been registered on ClinicalTrials.gov, ensuring transparency and accessibility of trial information.

The outcomes and findings of this study, whether positive or negative, will be disseminated through various channels to maximize their impact. Specifically, the results will be published in reputable peer-reviewed journals to contribute to scientific knowledge. Additionally, the findings will be shared with the broader medical community through presentations at national and international medical conferences. These dissemination efforts aim to promote transparency, scientific collaboration, and the application of research outcomes for the benefit of healthcare and patient well-being. Additionally, social media posts will be used to amplify the study findings and make the information more accessible to the lay public.

## Discussion

This paper presents the protocol for a RCT designed to evaluate if an ai-DPP is non-inferior to traditional h-DPP in reducing risk of type 2 diabetes in adults with overweight or obesity and prediabetes. This trial is innovative in its exploration of a digital health platform that leverages AI technology for delivering personalized, adaptive lifestyle coaching to individuals at risk of prediabetes, a concept rooted in JITAIs and behavioral change theory, without relying on human coaching. While preliminary feasibility and observational studies have indicated potential for JITAIs via mobile apps, their effectiveness compared to standard h-DPPs is yet to be established. This study, therefore, fills a significant gap in evidence for chronic disease prevention and health behavior modification.

The trial, which began in October 2021, faced initial challenges. These included discrepancies in participants’ baseline A1C levels compared to results of clinically obtained testing for prediabetes in the previous year, leading to an adjustment in inclusion criteria to align with CDC DPP entry requirements in which any laboratory criterion for prediabetes in the past year rendered the participant eligible to join. Despite this change, the majority of participants have a baseline A1C measurement in the prediabetes range, with a small subset joining the study on the basis of fasting glucose results or a clinically obtained A1C result in the prediabetes range. Additionally, technical issues with ActiGraph wrist monitors necessitated a switch to the ActiGraph CPIW for improved data synchronization and storage.

A key strength of this pragmatic clinical trial is its randomized controlled design in a real-world setting, enabling an intent-to-treat analysis, a contrast to most DPP outcome research which often relies on per-protocol analysis. This approach allows for a comprehensive understanding of DPP impacts in real-world clinical settings, considering various engagement barriers to both digital and human coach-based modalities. Furthermore, the study’s extensive data on physical activity in individuals with prediabetes, collected through frequent monthly measurements over a year, will enable detailed secondary analyses, filling a gap in objective physical activity measurement in this population.

Digital DPPs may offer advantages such as enhanced accessibility, scalability, consistent delivery, integrated data analysis, immediate feedback, and cost-effectiveness. However, they may lack personalization, struggle with adaptability, present challenges for technologically disadvantaged patients, and raise privacy concerns. Conversely, h-DPPs may excel in providing a personal touch, adaptive communication, complex decision-making, fostering trust and accountability, encouraging socialization, and have a well-established evidence base. Their limitations, however, lie in program availability, consistency, scalability, logistical complexities, and reimbursement [6, 46, 47] issues. Given that individual preferences may play an important role in selecting a digital vs. human coach-based DPP, a secondary objective will be to determine patient suitability for either intervention, assessed using the validated NPART survey [43]. This approach underscores the importance of personal preference in choosing between digital and human-led interventions in diabetes prevention.

This study is not without limitations. The nature of the interventions—comparing an AI with a human coach-based DPP—precludes the feasibility of a blinded design. To mitigate potential bias, objective measures are employed: weight and A1C levels are assessed by the research team rather than relying on self-reported data, and physical activity is measured objectively in the same way in both groups. Moreover, while the study team is aware of the treatment allocations, the biostatistician responsible for data analysis will remain blinded to these allocations. Aside from automated reminders regarding upcoming ActiGraph wear periods and phone calls prior to upcoming study visits, the research coordinators will not have any scheduled contact or coaching with the study participants. They are instructed to redirect any participant inquiries back to their respective DPPs.

It is also important to note that the primary endpoint is consistent with the CDC’s DPP recognition standards as of May 2021. However, any future changes in these guidelines might impact the comparability of our findings. Regarding the measurement of A1C levels, we employed two different methods. This approach aligns with the CDC’s methodology for determining A1C outcomes, although it might introduce some degree of variability. Lastly, the study examines the efficacy of a smartphone application, which, like all technology, is subject to ongoing updates and revisions. These changes could potentially alter the app’s functionality and user experience, thereby affecting the generalizability of our findings to future iterations of AI-based DPPs.

Despite these limitations, this trial is groundbreaking as it is, to our knowledge, the first large-scale, pragmatic clinical trial comparing an ai-DPP with standard of care. The outcomes of this study are poised to significantly influence diabetes prevention strategies, particularly in terms of scalability, drawing on prior evidence that fully digital DPPs can yield significant health benefits and cost savings by achieving comparable weight loss and engagement outcomes to traditional methods [17, 48, 49]. This could alleviate the burden of diabetes on healthcare systems and improve health outcomes for millions of individuals. A comprehensive cost-effectiveness analysis, incorporating both short-term and lifetime perspectives using a Markov model and probabilistic sensitivity analysis, will assess the economic viability of AI-based versus human coach-led diabetes prevention programs. This study addresses a critical gap in prospective evaluations of AI-based interventions for clinical effectiveness, especially in the context of just-in-time adaptive interventions and behavioral change, offering insights into the broader application of this approach for health behavior change. Our findings will be crucial for healthcare professionals, providing evidence-based support for integrating digital health platforms into their practice and empowering them with new tools to engage and support their patients in diabetes prevention.

## Trial status

Protocol version 1.8; August 14, 2023. Recruitment for this trial commenced in October 2021 and the final study participant was enrolled on December 20, 2023. Final study visits and data collection are anticipated to conclude by January 2025. The submission of this protocol was delayed beyond the completion of recruitment, primarily due to procedural modifications necessitated by the ActiGraph devices used in the trial. These changes, essential for ensuring the accuracy and reliability of physical activity data, required additional time for implementation and validation, thus impacting the timeline for protocol submission. It is important to note that the primary outcome remains consistent with the trial protocol as initially registered on ClinicalTrials.gov in September 2021. This delay in submission has not affected the integrity or the objectives of the trial.

### Supplementary Information

**Additional file 1.** Modified International Physical Activity Questionnaire short form (IPAQ-SF).

**Additional file 2.** Methods Used to Recruit Screened, Ineligible, and Enrolled Participants. Panel A. Recruitment method for all consented and screened individuals. Panel B. Recruitment method for ineligible (*N*=59) and enrolled (*N*=368) participants. Patient Portal = Epic Mychart (Johns Hopkins) or Epic MyTower (Reading). Social Media = Facebook ads. Provider = Referral from healthcare provider.

**Additional file 3.** Modified Physical Activity Readiness Questionnaire (PAR-Q).

## Data Availability

At the time of funding for this study, the National Institutes of Health (NIH) did not mandate data sharing. However, in the spirit of scientific collaboration and advancement, the authors are committed to considering the sharing of de-identified data. Upon completion of the study, it is our intention to deposit this data into a suitable repository, where it will be made accessible to other researchers upon reasonable request. Specific details regarding the repository and the process for accessing the data will be provided at a later stage, ensuring compliance with all ethical and legal standards for data sharing and participant privacy.

## Abbreviations

A1C: Hemoglobin A1C
AWS: Amazon Web Services
BMI: Body mass index
CDC: Centers for Disease Control and Prevention
CPIW: CentrePoint Insight Watch
d-DPP: Digital Diabetes Prevention Program
DPP: Diabetes Prevention Program
DSMB: The Data Safety Monitoring Board
EHR: Electronic health records
h-DPP: Human coach-based diabetes prevention programs
ai-DPP: Fully automated AI-powered digital diabetes prevention programs
ICER: Incremental cost-effectiveness ratios
ITT: Intention-to-treat
JHH: Johns Hopkins Hospital
JHU: Johns Hopkins University
JITAI: Just-in-time adaptive intervention
MDPP: Medicare Diabetes Prevention Program
MVPA: Moderate and Vigorous Physical Activity
NIDDK: The National Institute of Diabetes and Digestive and Kidney Diseases
PAR-Q: Physical Activity Readiness Questionnaire
QALY: Quality-adjusted life years
RCT: Randomized controlled trial
RDS: Relational data storage
REDCap: Research Electronic Data Capture
S3: Amazon’s Simple Storage Service
SPIRIT: Standard Protocol Items: Recommendations for Interventional Trials
SSL: Secure sockets layer
