# Supplementary figures and images for: Effectiveness of artificial intelligence vs. human coaching in diabetes prevention: a study protocol for a randomized controlled trial
Source: Trials. 2024 May 16;25:325. doi: 10.1186/s13063-024-08177-8 (PMC11100129; doi:10.1186/s13063-024-08177-8)

Additional file 1. Modified International Physical Activity Questionnaire short form (IPAQ-SF)


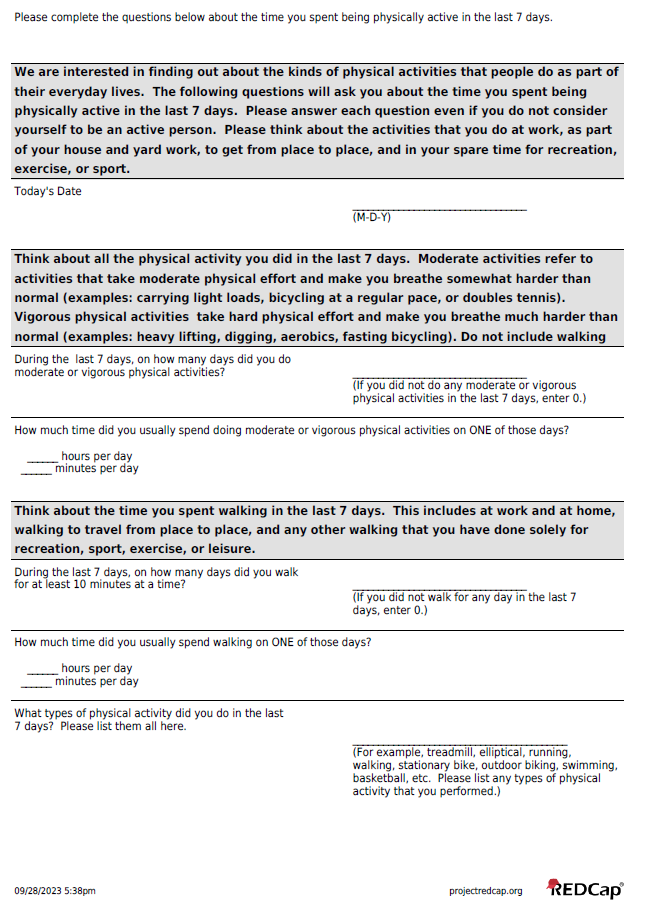

Supplement: Supplementary file 1 — Additional file 1. Modified International Physical Activity Questionnaire short form (IPAQ-SF). [file 13063_2024_8177_MOESM1_ESM.docx]
