# Supplementary material for: Effectiveness of artificial intelligence vs. human coaching in diabetes prevention: a study protocol for a randomized controlled trial
Source: Trials. 2024 May 16;25:325. doi: 10.1186/s13063-024-08177-8 (PMC11100129; doi:10.1186/s13063-024-08177-8)
Supplement: Supplementary file 2 — Additional file 2. Methods Used to Recruit Screened, Ineligible, and Enrolled Participants. Panel A. Recruitment method for all consented and screened individuals. Panel B. Recruitment method for ineligible (N=59) and enrolled (N=368) participants. Patient Portal = Epic Mychart (Johns Hopkins) or Epic MyTower (Reading). Social Media = Facebook ads. Provider = Referral from healthcare provider. [file 13063_2024_8177_MOESM2_ESM.docx]

## Additional file 2. Methods Used to Recruit Screened, Ineligible, and Enrolled Participants.


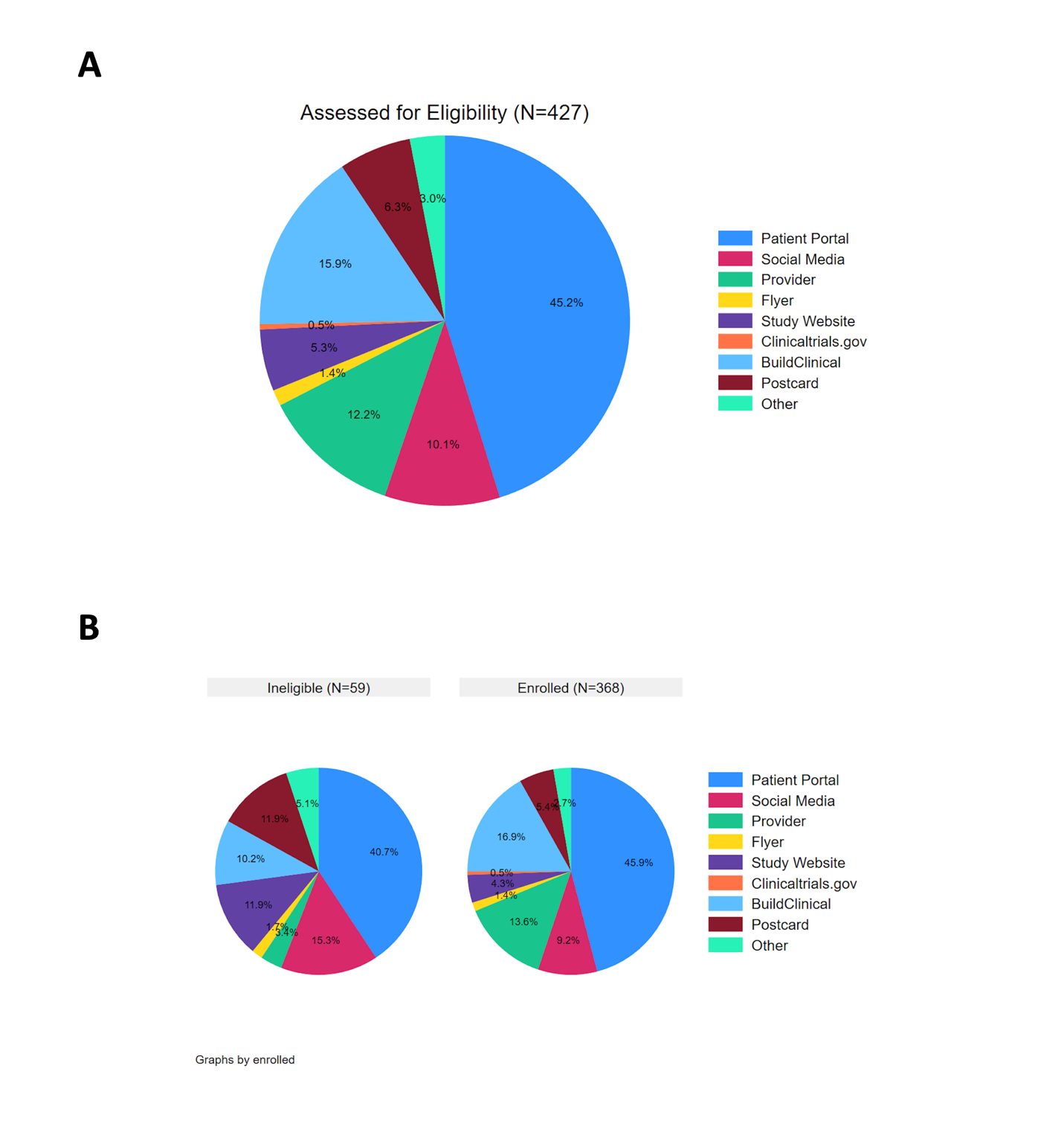


**Panel A.** Recruitment method for all consented and screened individuals. **Panel B.** Recruitment method for ineligible (N=59) and enrolled (N=368) participants. Patient Portal = Epic Mychart (Johns Hopkins) or Epic MyTower (Reading). Social Media = Facebook ads. Provider = Referral from healthcare provider.
