# Supplementary material for: Effectiveness of artificial intelligence vs. human coaching in diabetes prevention: a study protocol for a randomized controlled trial
Source: Trials. 2024 May 16;25:325. doi: 10.1186/s13063-024-08177-8 (PMC11100129; doi:10.1186/s13063-024-08177-8)
Supplement: Supplementary file 3 — Additional file 3. Modified Physical Activity Readiness Questionnaire (PAR-Q). [file 13063_2024_8177_MOESM3_ESM.docx]

# Additional file 3 . Modified Physical Activity Readiness Questionnaire (PAR-Q)

In this study, one goal will be to encourage participants to perform moderate levels of physical activity. The Diabetes Prevention Program is not an exercise program and promotes increases in moderate intensity physical activity (brisk walking, jogging, cycling, etc.), starting slowly and building up gradually. These questions will be used to determine whether it is appropriate for you to engage in moderate physical activity.

| 1. Has your healthcare provider ever said that you have a heart condition and that you should only do physical activity recommended by a healthcare provider? | Yes |
| --- | --- |
|  | No |
| 1. Do you feel pain in your chest when you do physical activity? | Yes |
|  | No |
| 1. In the past month, have you had chest pain when you were not doing physical activity? | Yes |
|  | No |

If participant answers **Yes** to one or more of the above questions, the study team will contact the primary care physician via electronic message or letter to obtain medical clearance prior to enrollment/randomization
